# *Diaporthe* species in south-western China

**DOI:** 10.3897/mycokeys.57.35448

**Published:** 2019-08-23

**Authors:** Hui Long, Qian Zhang, Yuan-Yuan Hao, Xian-Qiang Shao, Xiao-Xing Wei, Kevin D. Hyde, Yong Wang, De-Gang Zhao

**Affiliations:** 1 Department of Plant Pathology, College of Agriculture, Guizhou University, Guiyang, Guizhou 550025, China Guizhou University Guiyang China; 2 Guizhou Key Laboratory Agro-Bioengineering, Guizhou University Guiyang, Guizhou, 550025, China Sustainable Development Institute of Sandong Province Dongying China; 3 Guizhou Academy of Agricultural Sciences, Guiyang 550006, China Dejiang County Chinese herbal medicine industry development office Tongren China; 4 Administration Center of the Yellow River Delta Sustainable Development Institute of Sandong Province, Dongying, 257091, China Qinghai University Xining China; 5 Dejiang County Chinese herbal medicine industry development office, Tongren, 565200, China Mae Fah Luang University Chiang Rai Thailand; 6 Academy of Animal and Veterinary Sciences, Qinghai University (Qinghai Academy of Animal and Veterinary Sciences), Xining, China Guizhou University Guizhou China; 7 Center of Excellence in Fungal Research and School of Science, Mae Fah Luang University, Chiang Rai, 57100, Thailand Guizhou Academy of Agricultural Sciences Guiyang China

**Keywords:** *
Diaporthe
*, phylogeny, taxonomy, 2 new taxa

## Abstract

Three strains of the genus *Diaporthe* were isolated from different plant hosts in south-western China. Phylogenetic analyses of the combined ITS, *β*-tubulin, *tef*1 and calmoudulin dataset indicated that these strains represented three independent lineages in *Diaporthe*. *Diaporthe millettiae***sp. nov.** clustered with *D. hongkongensis* and *D. arecae*, *Diaporthe osmanthi***sp. nov.** grouped with *D. arengae*, *D. pseudomangiferae* and *D. perseae* and *Diaporthe* strain GUCC9146, isolated from *Camellia sinensis*, was grouped in the *D. eres* species complex with a close relationship to *D. longicicola*. These species are reported with taxonomic descriptions and illustrations.

## Introduction

Genus *Diaporthe* has been well-studied in recent years by Udayanga et al. (2011, 2012), incorporating morphological and molecular data and recommending appropriate genes to resolve species limitations in the genus. Since these revolutionary papers, 43 novel *Diaporthe* species have been described from China with morphological and phylogenetic evidence (Huang et al. 2013, 2015; Fan et al. 2016; Gao et al. 2014, 2015, 2016, 2017; Yang et al. 2017a,b, 2018; Yang et al. 2016; Du et al. 2016; Dissanayake et al. 2017a). Dissanayake et al. (2017b) provided an update of the genus with additional 15 species (7 new and 8 known species) from Italy based on molecular evidence. New records and species have also been reported by Hyde et al. (2016), Rossman et al. (2016), Chen and Kirschner (2017), Guarnaccia et al. (2018), Perera et al. (2018), Tibpromma et al. (2018) and Wanasinghe et al. (2018).

Three strains of *Diaporthe* were isolated from different medicinal plants collected in Guizhou and Guangxi during a survey of fungal diversity in south-western China. All the strains produced conidiomata containing alpha- and beta-conidia, typical of *Diaporthe*. This paper describes these three collections using molecular evidence, based on the analysis of combined ITS, *β*-tubulin, *tef*1 and calmoudulin datasets, as *Diaporthe millettiae* sp. nov. and *D. osmanthi* sp. nov. and *D. longicicola* with a new host record from *Camellia sinensis*.

## Materials and methods

### Isolation and morphological studies

The samples were collected from Guizhou and Guangxi provinces. The *Diaporthe* strains were isolated using the single-spore method (Chomnunti et al. 2014). Colonies, growing from single spores, were transferred to potato-dextrose agar (PDA) and incubated at room temperature (28 °C). Following 2–3 weeks of incubation, morphological characters were recorded as in Udayanga et al. (2011, 2015). Conidia and conidiophores were observed using a compound microscope (Olympus BX53). The holotype specimens are deposited in the Herbarium of Department of Plant Pathology, Agricultural College, Guizhou University (HGUP). Ex-type cultures are deposited in the Culture Collection at the Department of Plant Pathology, Agriculture College, Guizhou University, China (GUCC). Taxonomic information of the new taxa was submitted to MycoBank (http://www.mycobank.org) and Facesoffungi (http://www.facesoffungi.org).

### DNA extraction and sequencing

Fungal cultures were grown on PDA medium until they nearly covered the whole Petri-dish (90 mm diam.) at 28 °C. Fresh fungal mycelia were scraped from the surface with sterilised scalpels. A BIOMIGA Fungus Genomic DNA Extraction Kit (GD2416) was used to extract fungal genome DNA. DNA amplification was performed in a 25 μl reaction volume system which contained 2.5 μl 10 × PCR buffer, 1 μl of each primer (10 μM), 1 μl template DNA and 0.25 μl Taq DNA polymerase (Promega, Madison, WI, USA). Primers ITS4 and ITS5 (White et al. 1990) were used to amplify the ITS region. Three protein-coding gene fragments (*β*-tubulin, *tef*1 and calmoudulin) were amplified by the primers Bt2a/Bt2b (Glass and Donaldson 1995), CAL228F/CAL737R and EF1-728F/EF1-986R (Carbone and Kohn 1999). Gene sequencing was performed with an ABI PRISM 3730 DNA autosequencer using either a dRhodamine terminator or Big Dye Terminator (Applied Biosystems Inc., Foster 19 City, California). The sequences of both strands of each fragment were determined for sequence confirmation. The DNA sequences were submitted to GenBank and their accession numbers were provided in Table 1.

**Table 1.** GenBank accession numbers of isolates include in this study.

Species	Culture no.	GenBank no.			
		ITS	*tef*1	*β*-tubulin	calmoudulin
*Diaporthe alleghaniensis*	CBS 495.72	KC343007	KC343733	KC343975	KC343249
*D. ambigua*	CBS 114015	AF230767	GQ250299	KC343978	KC343252
*D. anacardii*	CBS 720.97*	KC343024	KC343750	KC343992	KC343266
*D. arecae*	**CBS 161.64**	KC343032	KC343758	KC344000	KC343274
*D. arengae*	**CBS 114979**	KC343034	KC343760	KC344002	KC343276
*D. baccae*	**CBS 136972**	KJ160565	KJ160597	MF418509	MG281695
*D. beilharziae*	**BRIP 54792**	JX862529	JX862535	KF170921	–
*D. betulae*	CFCC 50470	KT732951	KT733017	KT733021	KT732998
*D. bicincta*	CBS 121004	KC343134	KC343860	KC344102	KC343376
*D. biguttusis*	**CGMCC 3.17081**	KF576282	KF576257	KF576306	–
*D. celastrina*	**CBS 139.27**	KC343047	KC343773	KC344015	KC343289
*D. celeris*	**CBS 143349**	MG281017	MG281538	MG281190	MG281712
*D. charlesworthii*	BRIP 54884m*	KJ197288	KJ197250	KJ197268	–
*D. cinerascens*	CBS 719.96	KC343050	KC343776	KC344018	KC343292
*D. cotoneastri*	CBS 439.82	FJ889450	GQ250341	JX275437	JX197429
*D. decedens*	CBS 109772	KC343059	KC343785	KC344027	KC343301
*D. elaeagni*	CBS 504.72	KC343064	KC343790	KC344032	KC343306
*D. ellipicola*	**CGMCC 3.17084**	KF576270	KF576245	KF576291	–
*D. eres*	**CBS 138594**	KJ210529	KJ210550	KJ420799	KJ434999
*D. foeniculina*	**CBS 187.27**	KC343107	KC343833	KC344075	KC343349
*D. goulteri*	**BRIP 55657a**	KJ197289	KJ197252	KJ197270	–
*D. helianthi*	**CBS 592.81**	KC343115	GQ250308	KC343841	JX197454
*D. hongkongensis*	**CBS 115448**	KC343119	KC343845	KC344087	KC343361
*D. inconspicua*	**CBS 133813**	KC343123	KC343849	KC344091	KC343365
*D. longicicola*	GUCC9146	MK398676	MK480611	MK502091	MK502088
*D. longicicola*	**CGMCC 3.17091**	KF576267	KF576242	KF576291	–
*D. macinthoshii*	BRIP 55064a*	KJ197290	KJ197251	KJ197269	–
*D. millettia*	**GUCC9167**	MK398674	MK480609	MK502089	MK502086
*D. oncostoma*	CBS 589.78	KC343162	KC343888	KC344130	KC343404
*D. osmanthusis*	**GUCC9165**	MK398675	MK480610	MK502090	MK502087
*D. perseae*	CBS 151.73	KC343173	KC343899	KC344141	KC343415
*D. phragmitis*	**CBS 138897**	KP004445	–	KP004507	–
*D. pseudomangiferae*	**CBS 101339**	KC343181	KC343907	KC344149	KC343423
*D. pseudophoenicicola*	**CBS 462.69**	KC343184	KC343910	KC344152	KC343426
*D. rosicola*	MFLU 17.0646	NR157515	MG829270	MG843877	MG829274
*D. saccarata*	**CBS 116311**	KC343190	KC343916	KC344158	KC343432
*D. stitica*	CBS 370.54	KC343212	KC343938	KC344180	KC343454
*D. vaccinii*	CBS 160.32	AF317578	GQ250326	KC344196	KC343470
*Valsa ambiens*	CFCC 89894	KR045617	KU710912	KR045658	–

Ex-type isolates were labeled with bold.

### Phylogenetic analyses

DNA sequences from our three strains and reference sequences downloaded from GenBank (Dissanayake et al. 2017a, b), Guarnaccia et al. (2018) and Wanasinghe et al. (2018) were analysed by maximum parsimony (MP) and maximum likelihood (ML). Sequences were optimised manually to allow maximum alignment and maximum sequence similarity, as detailed in Manamgoda et al. (2012). MP analyses were performed in PAUP v. 4.0b10 (Swofford 2003), using the heuristic search option with 1,000 random taxa additions and tree bisection and re-connection (TBR) as the branch swapping algorithm. Maxtrees = 5000 was set to build the phylogenetic tree. The characters of the alignment document were ordered according to ITS+*tef*1+*β*-tubulin+CAL for GUCC9165 and GUCC9167 and *tef*1+*β*-tubulin for GUCC9146 with equal weight and gaps were treated as missing data. The Tree Length (TL), Consistency Indices (CI), Retention Indices (RI), Rescaled Consistency Indices (RC) and Homoplasy Index (HI) were calculated for each tree generated. The resulting Phylip file was used to make ML and Bayesian trees by the CIPRES Science Gateway (https://www.phylo.org/portal2/login.action) and RAxML-XSEDE with 1000 bootstrap inferences.

## Results

### Phylogenetic analyses

**Figure 1.**
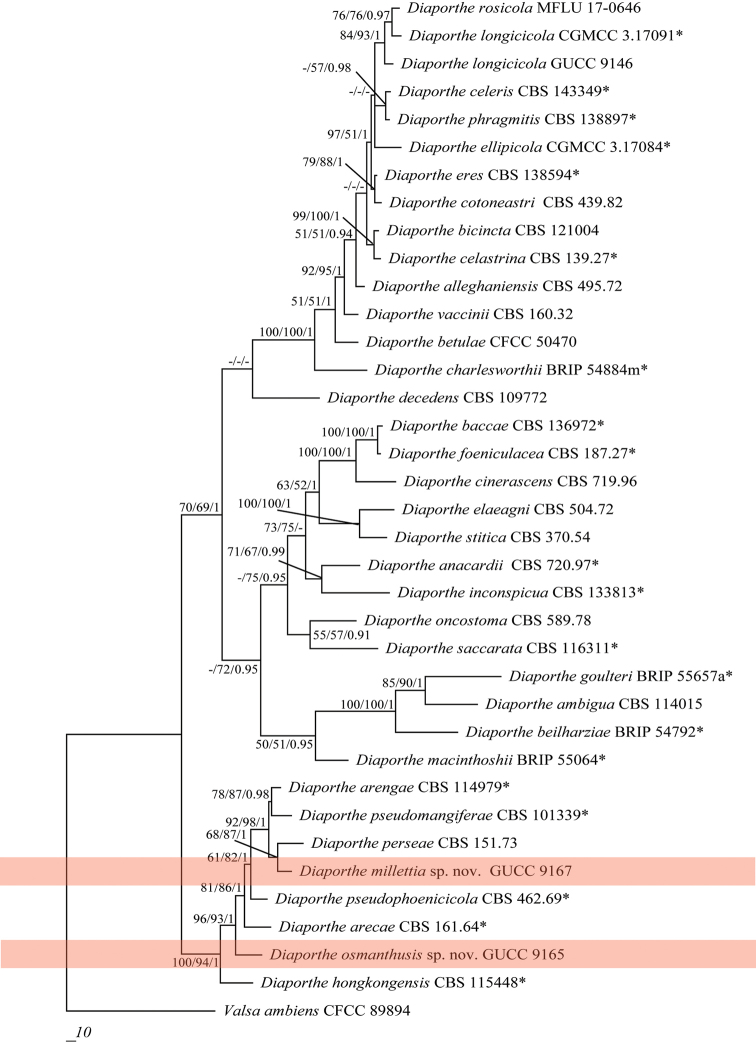
Parsimonious tree obtained from a combined analyses of an ITS, *β*-tubulin, calmoudulin and *tef*1 sequence dataset. MP, ML above 50% and BPP values above 0.90 were placed close to topological nodes and separated by “/”. The bootstrap values below 50% and BPP values below 0.90 were labelled with “-”. The tree is rooted with *Valsa ambiens* (CFCC89894). The branch of our new *Diaporthe* species is in pink.

**Figure 2.**
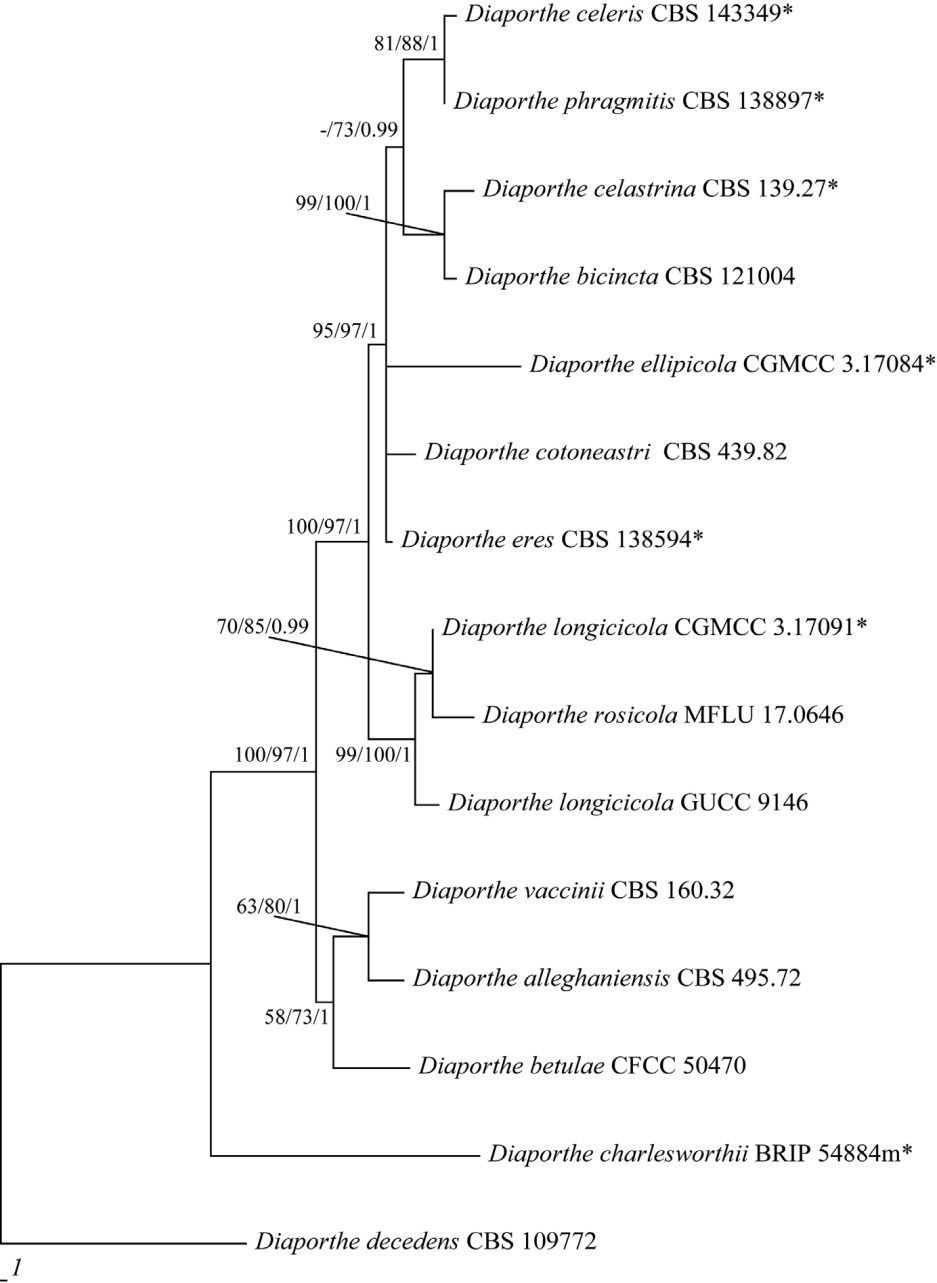
Parsimonious tree obtained from a combined analyses of a *β*-tubulin and *tef*1 sequence dataset (TL = 265, CI = 0.89, RI = 0.76, RC = 0.68 and HI = 0.11). MP, ML above 50% and BPP values above 0.90 were placed close to topological nodes and separated by “/”. The bootstrap values below 50% and BPP values below 0.90 were labelled with “-”. The tree is rooted with *Diaporthe decedens* (CBS 109772).

Three *Diaporthe* strains isolated from different plant hosts were sequenced. PCR products of 456–465 bp (ITS), 292–303 bp (*tef*1), 666–690 bp (*β*-tubulin) and 336–345 bp (CAL) were obtained. By alignment with the single gene region and then combination according to the order of ITS, *tef*1, *β*-tubulin and CAL with *Valsa ambiens* (CFCC 89894), only 1833 characters were obtained, viz. ITS: 1–492, *tef*1: 493–801, *β*-tubulin: 802–1469, CAL: 1470–1833, with 500 parsimony-informative characters. This procedure yielded eleven parsimonious trees (TL = 2169, CI = 0.58, RI = 0.71, RC = 0.41 and HI = 0.42), the first one being shown in Figure 1. All *Diaporthe* species clustered together, although without credible support for bootstrap and BPP values (Figure 1). Phylogenetic analysis of strains GUCC9165 and GUCC9167, using the four gene loci, confirmed them as well-resolved species (Figure 1). Strain GUCC 9165 formed an independent branch adjacent to *D. arecae* and *D. hongkongensis* (MP: 100%, ML: 94% and BPP: 1). Strain GUCC 9167 grouped with the branch which included *D. arengae*, *D. perseae* and *D. pseudomangiferae* (MP: 92%, ML: 98% and BPP: 1). Strain GUCC 9146 was aligned to the branch having *D. longicicola* and *D. rosicola* in the *Diaporthe eres* species-complex (Figure 2), with high statistical support (MP: 84%, ML: 93% and BPP: 1). This strain also showed a close relationship to *D. eres* and *D. cotoneastri*. In addition, we also compared the DNA base pair differences between our strains and related species in different gene regions (Suppl. material 1: Table S1). In *Diaporthe* strain GUCC9165, the four genes had 64 base pair differences with *D. arecae* and 119 with *D. hongkongensis*, the main differences being with *β*-tubulin and *tef*1. Strain GUCC9167 had 52 base pair differences with *D. arengae*, 61 with *D. perseae* and 64 with *D. pseudomangiferae*, wherein the base distinction was primarily in the *β*-tubulin and *tef*1 gene region. The *β*-tubulin sequences of *D. eres* and *D. longicicola* were apparently shorter than in strain GUCC 9146. The CAL sequences of *D. rosicola* were shorter than GUCC 9146. The DNA sequence of CAL for *Diaporthe longicicola* was not available (Gao et al. 2015). Integrating available DNA information, we discovered that 28 base pair differences were shown between GUCC 9146 and *D. eres*, 51 between GUCC 9146 and *D. cotoneastri*, 26 between GUCC 9146 and *D. rosicola* and 22 (only three genes) between GUCC 9146 and *D. longicicola*. Meanwhile, the phylogenetic analysis, based on only *tef*1 and *β*-tubulin for the *D. eres* species-complex (Figure 2), also indicated that GUCC 9146 clustered with *D. longicicola* and *D. rosicola* which obtained support values of MP: 99%, ML: 100% and BPP: 1 and maintained a closer relationship with *D. longicicola*.

### Taxonomy

#### Diaporthe millettiae

Taxon classificationFungiDiaporthalesDiaporthaceae

H. Long, K.D. Hyde & Yong Wang bis sp. nov.

18E3940B229E5A739D4216010B5CBA6D

MB829563

Figure 3

##### Diagnosis.

Characterised by larger J-shaped β-conidia.

##### Type.

China, Guangxi Province, Nanning City, from leaves of *Millettia reticulata*, 20 September 2016, Y. Wang, HGUP 9167, holotype, ex-type living culture GUCC 9167.

##### Description.

*Colonies* on PDA attaining 9 cm diam. after 10 days; coralloid with feathery branches at margin, adpressed, with apparent aerial mycelium, with numerous irregularly zonate dark stromata, isabelline becoming lighter towards the margin; reverse similar to surface, with zonations. *Conidiomata* pycnidial, multilocular, scattered, abundant on PDA after 3 wks, subglobose to irregular, 1.5–1.8 mm diam., ostiolate, with up to 1 mm necks when present. *Conidiophores* formed from the inner layer of the locular wall, sometimes reduced to conidiogenous cells, when present 1-septate, hyaline to pale yellowish-brown, cylindrical, 10–23 × 1–2.5 μm. *Conidiogenous cells* cylindrical to flexuous, tapered towards apex, hyaline, 8–18 × 1.5–3 μm. *Alpha conidia* abundant, fusiform, narrowed towards apex and base, mostly biguttulate, hyaline, 4.5–9 × 2–3.5 μm. *Beta conidia* scarce to abundant, flexuous to J-shaped, hyaline, 17.5–32 × 1–2 μm. *Perithecia* not seen.

##### Habitat and distribution.

Isolated from leaves of *Millettia reticulata* in China

##### Etymology.

Species epithet *millettiae*, referring to the host, *Millettia reticulata* from which the strain was isolated.

##### Notes.

Phylogenetic analysis combining four gene loci showed that *Diaporthe millettiae* (strain GUCC 9167) displayed a close relationship with *D. arengae*, *D. pseudomangiferae* and *D. perseae* with high bootstrap values (Figure 1). We compared the DNA base pair differences of the four gene regions, the main differences being in the *β*-tubulin and *tef*1 genes, especially *tef*1. *Diaporthe millettiae* produced two types of conidia (α, β), whereas *D. pseudomangiferae* only produced *alpha conidia* and *D. perseae* produced three types of conidia (α, β, γ). The β-conidia of *D. arengae* were smaller (20–25 × 1.5 μm) than those of *Diaporthe millettiae* (17.5–32 × 1–2 μm). The shape of β-conidia was also different. Conidiophores of *D. arengae* (10–60 μm) with more septa (0–6), were longer than those of *D. millettiae* (10–23 × 1–2.5 μm; 0-1-septate) (Gomes et al. 2013).

**Figure 3.**
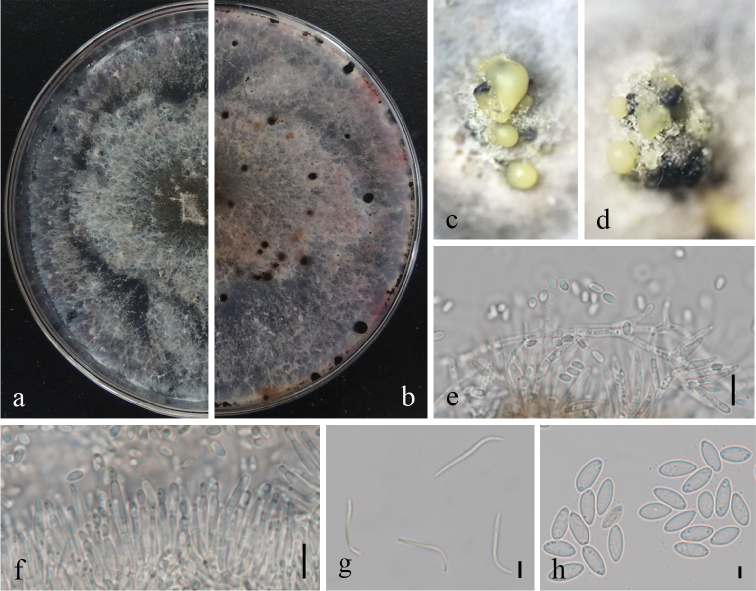
*Diaporthe millettiae* (GUCC9167). **a–b** upper (**a**) and lower (**b**) surface of colony on PDA**c–d** conidiomata **e–f** conidiophores, conidiogenous loci and conidia **g** β-conidia **h** α-conidia. Scale bars: 20 µm (**e, f**), 10 µm (**g, h**).

#### Diaporthe osmanthi

Taxon classificationFungiDiaporthalesDiaporthaceae

H. Long, K.D. Hyde & Yong Wang bis sp. nov.

AB53D9B53F1B5833915C42D14348045C

MB829564

Figure 4

##### Diagnosis.

Characterised by size of α-conidia and β-conidia.

##### Type.

China, Guangxi province, Nanning City, from leaves of *Osmanthus fragrans*, 20 September, 2016, Y. Wang, HGUP 9165, holotype, ex-type living culture GUCC 9165.

##### Description.

*Colonies* on PDA attaining 9 cm diam. after 10 days; coralloid with feathery branches at margin, adpressed, without aerial mycelium, with numerous irregularly zonated dark stromata, isabelline becoming lighter towards the margin; reverse similar to the surface with zonations more apparent. *Conidiomata* pycnidial and multilocular, scattered, abundant on PDA after 3 wks, globose, subglobose or irregular, up to 1–1.5 mm diam., ostiolate, necks absent or up to 1 mm. *Conidiophores* formed from the inner layer of the locular wall, reduced to conidiogenous cells or 1-septate, hyaline to pale yellowish-brown, cylindrical, 20.5–61 × 1–3 μm. *Conidiogenous cells* cylindrical to flexuous, tapered towards apex, hyaline, 10–15 × 1.5–3 μm. *Alpha conidia* abundant, fusiform, narrowed towards the apex and base, apparently biguttulate, hyaline, 5.5–8.5 × 2–3 μm. *Beta conidia* scarce to abundant, flexuous to J-shaped, hyaline, 20–31.5 × 1–2.5 μm. *Perithecia* not seen.

##### Habitat and distribution.

Isolated from leaves of *Osmanthus fragrans* in China.

##### Etymology.

Species epithet *osmanthi*, referring to the host, *Osmanthus fragrans* from which our strain was isolated.

##### Notes.

*Diaporthe osmanthi* (strain GUCC9165) formed an independent lineage, but was also related to *D. arecae* and *D. hongkongensis* (Figure 1). The sequences of *β*-tubulin and *tef*1 included about two-three differences between *D. osmanthi* (GUCC9165) and *D. arecae* (42) and *D. hongkongensis* (78) and thus they were different species according to the guidelines of Jeewon and Hyde (2016). Additionally, *Diaporthe hongkongensis* produced three types of conidia, but *Diaporthe osmanthi* did not produce γ-conidia. In addition, β-conidia of *D. hongkongensis* (18–22 μm) were shorter than those of *Diaporthe osmanthi* (Gomes et al. 2013). According to original description Srivastava et al. (1962), *D. arecae* also produced two types of conidia. The α-conidia (7.2–9.6 × 2.4 μm) were longer than in *Diaporthe osmanthi*, but its β-conidia (14.4–24 × 1.2 μm) were shorter and their shape also had some differences.

**Figure 4.**
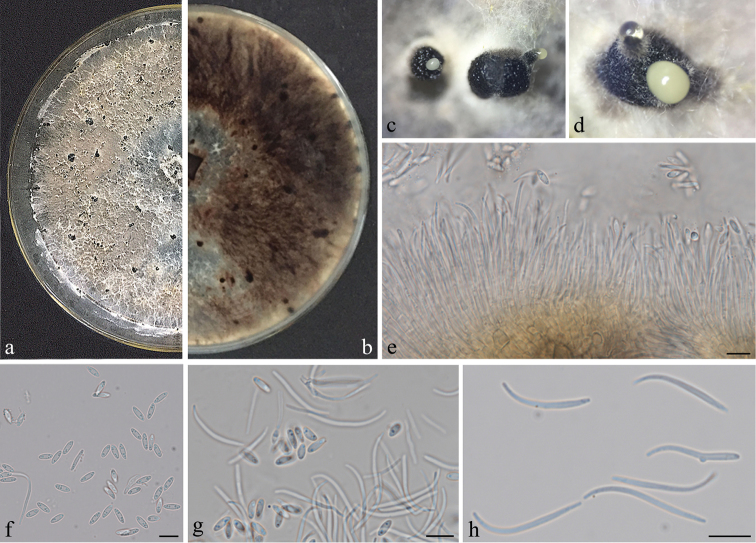
*Diaporthe osmanthi* (GUCC9165). **a–b** upper (**a**) and lower (**b**) surface of colony on PDA**c–d** conidiomata **e** conidiophores, conidiogenous loci and conidia **f** α-conidia **g** two types of conidia **h** β-conidia. Scale bars: 10 µm (**e, f, g, h**).

#### Diaporthe longicicola

Taxon classificationFungiDiaporthalesDiaporthaceae

Y.H. Gao & L. Cai, Fungal Biology 119(5): 303 (2015)

6147DDC1497558DB8A559F58761A6EC6

Figure 5

##### Description.

*Colonies* on PDA attaining 9 cm diam. in 10 days; coralloid with feathery branches at margin, adpressed, without aerial mycelium, without numerous irregularly zonated dark stromata, isabelline becoming lighter towards the margin; reverse similar to the surface with zonations more apparent. *Conidiomata* pycnidial and multilocular, scattered, abundant on PDA after 20 d, subglobose or irregular, 1.5–1.8 mm diam., ostiolate and up to 1 mm long. *Conidiophores* formed from the inner layer of the locular wall, densely aggregated, hyaline to pale yellowish-brown, cylindrical, tapering towards the apex, 15–25 × 1.5–2 μm. *Alpha conidia* abundant, ellipsoid to fusiform, apparently biguttulate, hyaline, 6–9 × 2–3 μm. *Beta conidia* scarce to abundant, flexuous to J-shaped, hyaline, 25.5–35.5 × 1–2.5 μm.

##### Habitat and distribution.

Isolated from leaves of *Camellia sinensis* in Duyun, Guizhou Province, China

##### Notes.

Phylogenetic analyses (Figures 1, 2) indicated that GUCC 9146 has a close relationship with *D. longicicola*, *D. rosicola*, *D. eres* and *D. cotoneastri*. Morphological comparison indicated that this strain was most similar to *D. longicicola* but not a related species by the width of alpha conidia and length of beta conidia (Udayanga et al. 2014; Gao et al. 2015).

**Figure 5.**
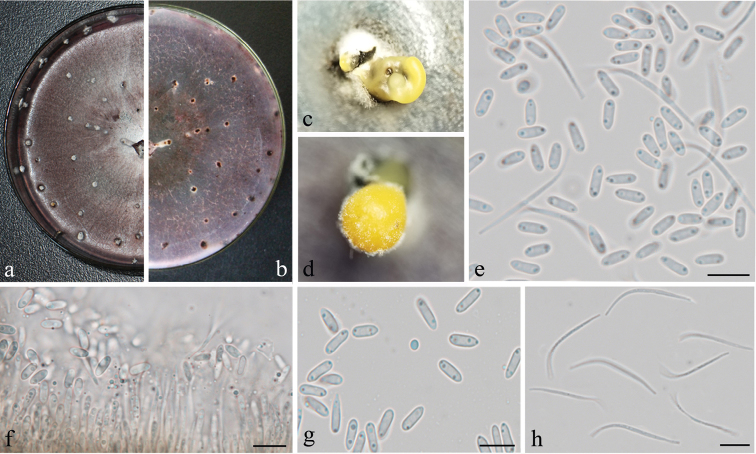
*Diaporthe longicicola* (GUCC9146). **a–b** upper (**a**) and lower (**b**) surface of colony on PDA**c–d** conidiomata **e** two types of conidia **f** conidiophores, conidiogenous loci and conidia **g** α-conidia **h** β-conidia. Scale bars: 10 µm (**e, f, g, h**).

## Discussion

Phylogenetic analysis and morphology provide evidence for the introduction of *Diaporthe millettiae* and *D. osmanthi* as new species. In order to support the validity of these new species, we followed the guidelines of Jeewon and Hyde (2016) in comparing base pair differences (Suppl. material 1: Table S1). In accordance with Udayanga et al. (2014), we also believed that the ITS fragment was problematic for the *D. eres* species-complex. When not considering ITS, integration with morphological comparison was helpful and we concluded that GUCC 9146 is *D. longicicola. Diaporthe longicicola* was firstly reported on *Lithocarpus glabra* in Zhejiang Province, but our strain (GUCC 9146) was recovered from *Camellia sinensis* in Guizhou Province. Thus, this is the report of a new host and new location in China for *D. longicicola*.

## Supplementary Material

XML Treatment for Diaporthe millettiae

XML Treatment for Diaporthe osmanthi

XML Treatment for Diaporthe longicicola

## Supplementary materials

Supplementary material 1 The DNA bases difference between our strains and related taxa on four gene regions Data type: molecular data https://binary.pensoft.net/file/328116This dataset is made available under the Open Database License (http://opendatacommons.org/licenses/odbl/1.0/). The Open Database License (ODbL) is a license agreement intended to allow users to freely share, modify, and use this Dataset while maintaining this same freedom for others, provided that the original source and author(s) are credited.Hui Long, Qian Zhang, Yuan-Yuan Hao, Xian-Qiang Shao, Xiao-Xing Wei, Kevin D. Hyde, Yong Wang, De-Gang Zhao
