# Supplementary material for: Diaporthe species in south-western China
Source: MycoKeys. 2019 Aug 23;57:113–27. doi: 10.3897/mycokeys.57.35448 (PMC6717119; doi:10.3897/mycokeys.57.35448)
Supplement: Supplementary material 1 [file mycokeys-57-113-s001.docx]

**Table S1**. The DNA bases difference between our strains and related taxa on four gene regions.

| Species | Strain number | ITS (462) | β-tubulin  (628) | *tef*1  (249) | CAL  (393) |
| --- | --- | --- | --- | --- | --- |
|  | GUCC 9167 | 0 | 0 | 0 | 0 |
| *D. arengae* | CBS 114979 | 4 | 16 | 22 | 10 |
| *D. perseae* | CBS 151.73 | 14 | 11 | 26 | 10 |
| *D. pseudomangiferae* | CBS 101339 | 6 | 23 | 28 | 9 |
| Species | Strain number | ITS (462) | β-tubulin  (647) | *tef*1  (250) | CAL  (391) |
|  | GUCC 9165 | 0 | 0 | 0 | 0 |
| *D. hongkongensis* |  | 20 | 40 | 38 | 21 |
| *D. arecae* |  | 11 | 24 | 18 | 11 |
| Species | Strain number | ITS (465) | β-tubulin  (653) | *tef*1  (245) | CAL  (346) |
|  | GUCC 9146 | 0 | 0 | 0 | 0 |
| *D. longicicola* |  | 17 | 3 | 2 | --- |
| *D. rosicola* |  | 7 | 8 | 6 | 5 |
| 1. *eres* |  | 10 | 16 | 1 | 1 |
| *D. cotoneastri* |  | 10 | 16 | 2 | 23 |
